# Using photos of basic facial expressions as a new approach to measuring implicit attitudes

**DOI:** 10.1371/journal.pone.0250922

**Published:** 2021-05-13

**Authors:** Klara Malinakova, Richard Korinek, Peter Tavel, Iva Polackova Solcova, Harold G. Koenig, Jitse P. van Dijk, Sijmen A. Reijneveld

**Affiliations:** 1 Palacký University Olomouc, Olomouc University Social Health Institute, Olomouc, Czech Republic; 2 Department of Community and Occupational Medicine, University of Groningen, University Medical Center Groningen, Groningen, The Netherlands; 3 Czech Academy of Sciences, Institute of Psychology, Prague, Czech Republic; 4 Department of Medicine and Psychiatry, Duke University Medical Center, Durham, North Carolina, United States of America; 5 Department of Medicine, King Abdulaziz University, Jeddah, Saudi Arabia; 6 Graduate School Kosice Institute for Society and Health, P.J. Safarik University in Kosice, Kosice, Slovak Republic; National Institutes of Health, UNITED STATES

## Abstract

**Background:**

Measuring implicit attitudes is difficult due to social desirability (SD). A new method, the Emotion Based Approach (EBA), can solve this by using emotions from a display of faces as response categories. We applied this on an EBA Spirituality tool (EBA-SPT) and an Actual Situation tool (EBA-AST). Our aim was to assess the structure, reliability and validity of the tools and to compare two EBA assessment approaches, i.e., an explicit one (only assessing final replies to items) and an implicit one (assessing also the selection process).

**Methods:**

We obtained data on a sample of Czech adults (n = 522, age 30.3±12.58; 27.0% men) via an online survey; cortisol was assessed in 46 participants. We assessed the structure and psychometric properties (internal consistency and test-retest reliability; convergent, discriminant, and criterion validity) of the EBA, and examined the differences between explicit vs. implicit EBA approaches.

**Results:**

We found an acceptable-good internal consistency reliability of the EBA tools, acceptable discriminant validity between them and low (neutral expression) to good (joy) test-retest reliability for concrete emotions assessed by the tools. An implicit EBA approach showed stronger correlations between emotions and weaker convergent validity, but higher criterion validity, than an explicit approach and standard questionnaires.

**Conclusion:**

Compared to standard questionnaires, EBA is a more reliable approach for measuring attitudes, with an implicit approach that reflects the selection process yielding the best results.

## Introduction

Measuring attitudes related to deeply personal topics, issues and convictions, e.g., religiosity and spirituality (R/S), is a challenge in psychological and sociological research [1]. Measurement errors are particularly common, with social desirability bias (SDB) being a major issue. This could explain why many studies fail to find significant associations between psychological variables and various biomarkers. For example, cortisol is a stress hormone that can be measured in saliva and is used as a validity criterion for various associations with stress. However, in their review, Campbell and Ehlert [2] found that significant correlations between cortisol responses and perceived emotional stress variables were found in only 25% of the studies and a more recent study by Reyes-Ortiz, Berges [3] didn’t find any association of an artificially elevated cortisol level with self-reported stress. Evidently, the measurement of psychological variables can be improved.

The challenge of SDB, i.e., the tendency of individuals to present themselves in a more favorable light [4], is particularly likely to occur in the measurement of R/S. Zerbe and Paulhus [5] distinguish two components of SDB: impression management and self-deception. Impression management represents the conscious presentation of false answers and to a certain degree could be addressed by ensuring the anonymity of respondents [6]. Self-deception might be more difficult to address, as the participants believe the false information they report [5] and might not even be aware of their deeper feelings [7]. In measuring R/S, social desirability in particular affects images of God, with a likely discrepancy between one’s rational idea of God and deeper emotional feelings [8]. Innovative solutions are needed to assess these feelings.

One solution may be to use alternative approaches that avoid verbal answers, as some respondents may find it difficult to reach deeper emotional experiences cognitively and to express them verbally. Various implicit approaches could help to deal with this, e.g., the Implicit Attitude Test [9], and projective techniques which try to assess the construct of interest without asking directly for a verbal report [10]. These so-called enabling techniques could yield more reliable measurements of people’s attitudes. Moreover, they can help lower the effect of different sociocultural expectations that may lead to suppression of certain emotions that might be considered unacceptable [11]. Though definitely promising, most enabling techniques require a trained administrator to assess the outputs which makes them inconvenient for large-scale research.

Thus, another potential solution that could be helpful in large-scale applications regards the use of various non-verbal pictorial assessment techniques, e.g., drawings of human faces differing in the shapes of the mouth and sometimes eyebrows, instead of classical verbal answers. This approach has already been used to measure attitudes to work [12] and various life domains [13]. Other examples are the Faces scale [14] and the Self-Assessment Manikin [15], which measure the pleasure, arousal and dominance associated with a person’s affective reaction to different stimuli. An extension of this idea could be to use photographs of human faces which may be closer to the real emotional experience of a person. Moreover, the above-mentioned scales used only a single “joy—neutral—sadness/anger” dimension, which may negatively influence the validity of a measure if other emotions were predominant. For example, if a participant’s main emotion is fear, it might be difficult for the respondent to identify with any of the options. Therefore, an alternative to classical questionnaires that includes the above-mentioned findings could be the use of a multidimensional tool, i.e., the assessment of the participants’ responses to simple verbal stimuli (tool items) via choosing a corresponding photo of a basic facial expression from a display of emotions as a response category. Besides using this approach in R/S assessment, we also decided to explore the stress in one’s current life, because it allows the use of cortisol assessment as criterion validity.

Therefore, the aim of this study was to explore whether our new method, the Emotion Based Approach (EBA), specifically the EBA-Spirituality tool and the EBA-Actual Situation tool, represent a reliable alternative to classical questionnaires (Daily Spiritual Experience Scale, Brief Symptom Inventory). We assessed the structure (correlations between the emotions, descriptive statistics) and psychometric properties (internal consistency and test-retest reliability; convergent, discriminant, and criterion validity) of the EBA, and examined whether the results differed for an explicit vs. an implicit EBA approach.

## Methods

### Participants

The sample size for the study was determined with a power analysis conducted in R "pwr" package using an alpha of 0.05 and a power of 0.80. According to Bonett and Wright [16], for medium effect size of r = 0.3 and alpha = 0.05, the sample size that yields a Fisher confidence interval having the width of 0.3 is n = 149. Based on these results, a sample of 150 respondents was considered to be sufficient for most of the assessment, including the test-retest evaluation.

Altogether, we obtained data on a sample of 651 Czech respondents aged 15 years and over (December 2016-December 2017) using a snowball technique; 109 participants also gave saliva for cortisol determination. The median time for filling in the online survey was 33 minutes. Eleven respondents were excluded from it because of the extremely short time filling in the survey (i.e., less than 15 minutes), which did not allow them to fill in the survey thoughtfully. This led to a sample of 640 respondents (mean age 30.0, SD = 12.25; 25.5% men), in which 200 of these also completed the retest study. As described in detail in the EBA development and procedure, these 640 respondents involved both a pilot study sample and a sample for the main analysis. The results of the pilot analyses are attached as S3 File–Results of the pilot study, therefore, throughout this study we describe only the results of the analyses performed on the main sample, which consisted of 522 respondents (mean age 30.3, SD = 12.58; 27.0% men).

For the cortisol assessment, the inclusion criteria were attendance at a university and age within the range of 18–28 years. The exclusion criteria were: recent abuse of any illegal addictive substance (6 months), pregnancy or breast-feeding, endocrine problems, shift work and mouth redness due to infection or injury or an unusually high level of stress on the day of the experiment. All these criteria were assessed using self-reported questions, e.g., the question on the level of stress was worded as follows: In terms of stress, these days are for you: with the following response options: a) abnormally peaceful; b) normal; c) more stressful than normally.

All the respondents who took part in this assessment decided on their participation based on a detailed information sheet that described the aim and nature of the study, the inclusion and exclusion criteria for participation, and the saliva collection procedure. Altogether, saliva samples from 109 respondents were analyzed, however, in the main text of the article we present only the results of the analyses performed on a smaller subsample of 46 respondents (mean age 21.2, SD = 2.02 years; 32.6% men) for which the final version of the tool was used. For the analyses on the pilot sample please see S3 File–Results of the pilot study.

The study design was approved by the Ethics Committee of the Olomouc University Social Health Institute, Palacký University Olomouc (No. 2016/3). Participation in the survey was fully voluntary, so the respondents could stop participating in the survey at any time. Given the online nature of the survey, it was not possible to obtain written informed consent from each participant. Therefore, respondents expressed the agreement with their participation in the study by checking the corresponding box before the beginning of their online survey.

### Procedure and measures

#### EBA (Emotion Based Approach) method

The EBA method is an approach that assesses reactions to simple questionnaire items through the choice of a corresponding facial expression from a display of 13 pictures with human faces (for detailed introductory instructions and concrete sets of items please see the S1 File–Description of the EBA tools). These pictures standardly depict various emotions (a neutral face and two degrees of expression of each of the basic emotions, a weak one and a strong one). The items can be designed according to different research areas. The main requirements for designing new items are that they are simple and do not invoke specific emotions merely by their formulation. For scoring purposes, each of the basic emotions (joy, anger, fear, disgust, sadness, surprise, and a neutral emotion) represents a unique answering category. In the set of 13 pictures that we used for the assessment, each emotion is expressed either weakly or strongly and there is one picture only for the neutral face. Assuming that the intensity of emotion experienced is linearly related to the facial expression stimulus, we decided to distinguish between its two degrees. We have assessed all the pictures using the FaceReader, facial expression recognition tool (Noldus, version 7.1). As the weak expression of all the emotions was in general close to the neutral expression, we have scored them all with one point. Given the fact that the degree of emotional expression was not standardized (this is only possible for computer generated faces) and thus its intensity fluctuated for every emotion, we have simplified the procedure and have scored any strong expression of an emotion with two points. The score for each emotion was then aggregated over the tool and/or tool set of items. Next, we also registered the characteristics of the way of responding, leading to explicit and implicit responses. An explicit EBA approach assesses only the final replies to the items. We used an online tool; however, it could possibly be realized also via paper-pencil administration. An implicit approach also assesses the selection process in the sense of which other emotions a participant looked at (i.e., enlarged from the display) before choosing a final one; this requires an online tool, as described below.

#### EBA development and procedure

As a specific application of these principles, we developed and tested an EBA Spirituality Tool (EBA-SPT), i.e., a specific EBA application to measure R/S. It consists of two sets of questions based on contemporary research in R/S: the first set of items focuses on non-religious spirituality (NRS) and contains the following items: meaning of life, me and the world, my past, my future, my spiritual life, the aim of my life, forgiveness, engagement for others. The second set of items focuses on the God-Image (GI) and contains the following items: God, prayer, God’s will, God’s closeness, I can hear God talking about me, meeting with God at the end of my life, alone with God, God in my life.

For the purpose of designing a tool for measuring possible actual stress levels in different areas of life, the EBA Actual Situation tool (EBA-AST) was also created and used as an additional set of items, which were designed to cover the main possible sources of everyday stress. It contains the following items: how am I, today, people around me, my life, my work, my relationships, my needs, my health.

**Fig 1:**
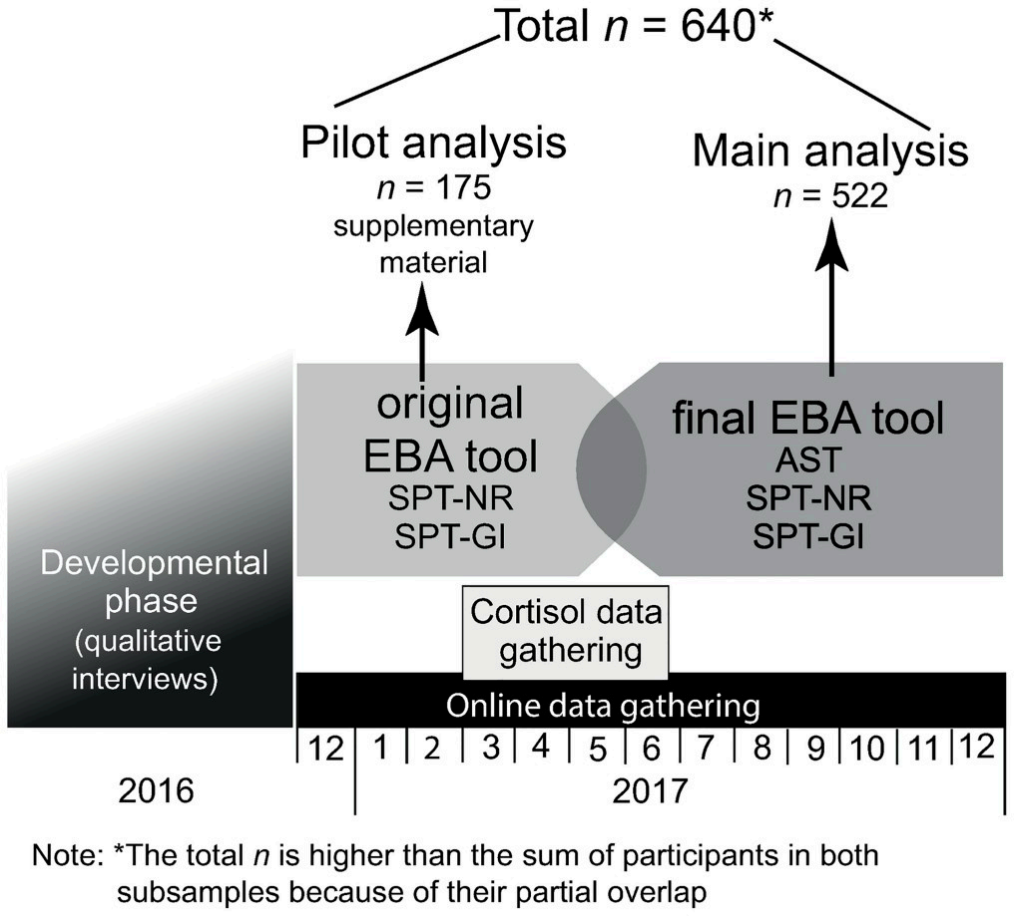
Timeline of the study.

The phases of the development and testing of the EBA tool are depicted in Fig 1. In the development of the EBA tool, we first tested our original idea using qualitative interviews (developmental phase). This regarded 28 in-depth interviews (lasting 60–180 minutes) in which participants, besides answering other questions on their spirituality and their relationship to God, also responded to items of the EBA-SPT by choosing a corresponding facial expression on the first version of the display of emotions. In this task, we used an A4-sized page with a table consisting of 49 prototypical faces, each displaying a different emotion/combination of emotions, as published by Vanger et al. [17]. We measured how quickly the participants were able to give an answer by choosing a facial expression and how much this choice corresponded to their consequent report about their spirituality and their relationship to God.

In general, interviews in the developmental phase suggested that participants were able to answer the questions by choosing a picture. However, it also showed that sometimes their immediate choice was more positive than their consequent deeper commentary, which also often mirrored negative emotions towards God. This discrepancy has already been observed by other authors [8] and is believed to be associated with SDB. Therefore, in order to find a way to reduce SDB, we developed an online version of the EBA tool which could record a final choice of a participant, but also the whole selection process. We believed that as participants were not aware of this additional monitoring, the SDB in this step could be lowered.

In this online version of the survey, as in the developmental phase, we used EBA-SPT. For every EBA item the pictures were displayed in random order in three rows and their position changed upon every page reload. The EBA tool recorded mouse movements over individual pictures. After hovering with the cursor over a picture for longer than 800ms the selected picture was enlarged and raised above the others, which was recorded as a “hover”. After clicking on the enlarged picture, it turned into a selection and the face was displayed in a dialog window, which could be either submitted or dismissed. This event was recorded as a “display”. When submitted, it was recorded as a “selection” and the next item was then displayed. For every item we collected the number of hover counts, display counts and selection counts per emotion, finally leading to two measures: selection counts (SC), representing an explicit EBA approach, and counts of hovers and of displays (hover-display count, HDC), representing an implicit EBA approach. The survey was hosted on virtual servers provided by Palacký University Olomouc, Czech Republic. The front-end was implemented as an interactive web page using standard Bootstrap layout. The system was secured by the Google reCaptcha system to avoid abuse of the system by bots.

As a next step (pilot analysis), we started to spread the online EBA-SPT among Czech adults using a snowball technique and we asked university students to participate in a cortisol assessment. Participants completed the survey, which included the EBA-SPT (non-religious respondents filled in only the EBA-SPT-NRS) and consequently used a generated code for entering their retest results. For the cortisol assessment study, they entered the system with pre-distributed access codes.

We checked the functioning of the pilot version of EBA-SPT during the first part of the data collection. This evaluation of the pilot survey results showed some associations of the EBA-SPT with the cortisol levels (see S3 File–Results of the pilot study); however, it brought up questions about the usability of the first display of emotions, because the emotions were not graded by their intensity but displayed as combinations of upper and lower face parts with different facial expressions, which is not natural and potentially confounding. Therefore, while continuing to gather data using the pilot version of the EBA, we also added into the survey a new version of the tool, which was finally used for the main analyses presented in this study. Instead of the original table, this new version used a new table composed of natural photographs of human emotions from the Emotions Revealed Photo Set (Paul Ekman Group). Using this new version of the EBA, we collected 522 respondents, 57 of whom filled in both versions of the EBA-SPT for comparison purposes. Of all the respondents, 109 participated in the cortisol assessment; however, for the main analysis we used only a subsample of 46 respondents who filled in the new version of the EBA tool. Cortisol analyses using a pilot version of the EBA tool are presented separately as S3 File–Results of the pilot study.

Moreover, in order to improve the use of cortisol as criterion validity and to avoid simultaneous testing of several assumptions (e.g., the associations of spirituality with cortisol levels), we have developed an additional tool to measure the cumulative effect of possible sources of stress that could influence a participant’s cortisol levels on the day of the experiment: the EBA Actual Situation Tool (EBA-AST). It contains the following items: how am I, today, people around me, my life, my work, my relationships, my needs, my health. These items were designed to capture the current situation of the respondent on the days of the experiment (how am I, today) as well as other possible sources of everyday stress. The numbers of respondents in each assessment are summarized in Table 1. In the whole survey, all the EBA tools were incorporated at its beginning, following the questions on basic socio-demographic characteristics and questions to assess eligibility for cortisol assessment.

**Table 1:** Summary of the numbers of participants in the whole sample and concrete subsamples.

	Total sample	Test-retest subsample	Cortisol subsample
Pilot version			
EBA-SPT-NRS	175	54	103
EBA-SPT-GI	166	54	103
Final version			
EBA-AST	522	146	46
EBA-SPT-NRS	522	146	46
EBA-SPT-GI	436	113	40
Total	640	200	109

All of the further analyses in this study are presented on the subsample of 522 respondents answering through a final version of the tool. For a trial version of the final EBA tool, please see https://dotaznik.oushi.upol.cz/questionnaire/start/7/broker/web/assignment/6. http://dotaznik.korinek.link/questionnaire/start?id=7

#### Test-retest analysis

Together with implementing a second version of the EBA tool, we also started a new test-retest survey. The participants who filled in the main survey were also asked to fill in a test-retest one to two weeks after the first survey. This timeframe was chosen because we assumed that the participants would not remember their choices and that the responses would reflect their actual mood at that moment. This retest in the main study was completed by 146 respondents.

#### Criteria for validity

For convergent validity, we used the Brief Symptom Inventory (BSI-53) and Daily Spiritual Experience Scale (DSES) as gold standards. The Brief Symptom Inventory (BSI-53) measures psychological symptoms [18]. It consists of 53 items. The BSI was scored and profiled in terms of nine subscales, i.e., eight primary symptom dimensions and the Global Severity Index (GSI) measuring the overall psychological distress level. The tool was validated in the Czech environment [19] and Cronbach’s alpha for the GSI was 0.97 in our sample.

The Daily Spiritual Experience Scale (DSES) measures the frequency of ordinary experiences of connection with the transcendent in daily life [20]. An adapted, 15-item version [21] of the scale was used in this study. The scale was administered only to a part (319 of 522) of the respondents. Cronbach’s alpha was 0.91 in our sample.

For criterion validity we used the Dopen Questionnaire Lie Score and the measured salivary stress hormone cortisol as gold standards. As the criterion validity, we used the Dopen Questionnaire Lie Score and measured cortisol levels. The Dopen Questionnaire Lie Score [22] consists of 14 questions assessing one’s tendencies to socially desirable responding. In this study we used 13 questions. Cronbach’s alpha was 0.73 in our sample.

With regards to the measurement of cortisol levels, participants were instructed not to use an illegal drug or ingest a large amount of alcohol 48 hours prior to providing the sample and to acquire the first sample of cortisol immediately after getting up, by 8:00 a.m. at the latest. They were further instructed not to eat, drink, smoke, clean their teeth or use dental floss and to remove lipstick or lip balm. Prior to the experiment, participants collected a set of two Salivettes with the blue cap (Sarstedt, Nümbrecht, Germany) on a special dispensing point. Each pair of Salivettes was already labelled with a randomly generated code. On the day of the experiment, the participants took two saliva samples, the first one immediately after waking-up and the second one 30 minutes after the first one. The participants chewed on a synthetic swab for 1 minute. Afterwards, the swabs were placed in the plastic tube of the Salivettes. On the same day, by noon, they then filled-in the online survey. When they opened the online survey and expressed their agreement with participation in the study, they could use the code on their Salivettes for entering into the survey. Consequently, they delivered Salivettes with their saliva samples back to the same dispensing point. The samples were either stored in a refrigerator and analyzed within 3 days, or stored at -20°C until they were analyzed. Biochemical analyses were performed at the Department of Clinical Biochemistry of the University Hospital in Olomouc using an ELISA kit (Salimetrics, State College, PA). Free cortisol levels have been shown to increase rapidly within the first 30 minutes after awakening [23]. We therefore measured the baseline and the 30 minute follow-up levels. Based on these measurements, we calculated cortisol reactivity, i.e., the change in cortisol levels between the baseline and follow-up.

#### Background and control questions

We further obtained data on the background of the respondents and asked control questions regarding the EBA-SPT, EBA-AST and cortisol assessments. The background characteristics regarded gender, age and other basic sociodemographic characteristics (marital status, highest education achieved, religiosity).

Control questions on reading emotions were placed at the end of the EBA questionnaire and were based on pictures depicting three strong expressions of emotions, which were preceded by an introductory statement: “According to your opinion, this girl is most likely to:”. For each question, a picture was shown with one of these basic emotions strongly expressed, and the participants had to choose the right answer of three options describing a situation that the person might be experiencing (please see S2 File–Control questions for the concrete wording of the items and for the comparison of groups of respondents who were able to recognize emotions and those who were not). The participants who answered all three items correctly were considered as being able to recognize emotions, the rest as not being able to do so. However, as the preliminary assessment did not validate discarding respondents who were unable to recognize emotions, we present in this study the results based on the assessment of both groups.

The control questions for cortisol assessment included the perceived level of actual stress, recent (48 hours) abuse of alcohol or an illegal drug, recent (6 months) dependence on any illegal drug, endocrine problems, use of steroids, phase of the menstrual cycle and recent (one month) use of oral contraceptives.

### Statistical analyses

**Fig 2:**
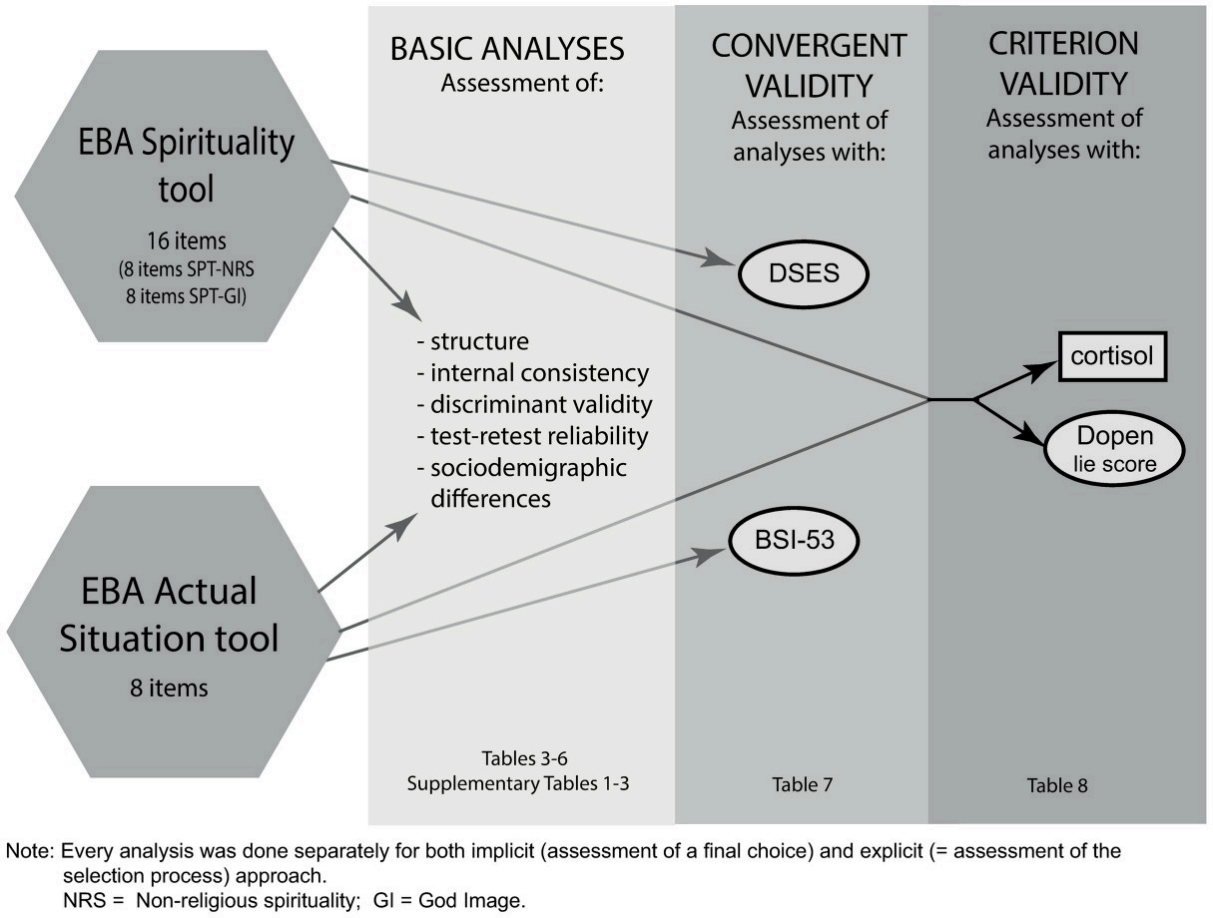
Description of the EBA tools and statistical analyses.

First, we described the background characteristics of the sample. Next, we assessed the EBA-SPT and EBA-AST based on the conceptual model, as shown in Fig 2. Each of the further steps was done in parallel for an explicit (SC) and an implicit (HDC) EBA approach.

In the second step, we assessed the structure of the EBA tools using Spearman’s rank order correlation (r_s_) between the scores of all basic emotions, as assessed by the EBA tools. Third, we tested the psychometric properties (reliability and validity) of the EBA tools. The reliability of the EBA tools and their sets of items was assessed using Cronbach’s alpha (α) based on Standardized Items and Mean Inter-Item Correlation values (MIIC). As the nature of the tool does not allow the standard item-by-item assessment, we had to choose an alternative approach for measuring internal consistency: based on the previous analysis of the EBA structure (see Results section, Table 2), emotions were scored as follows: -1 point for a weak expression of joy, -2 points for a strong expression of joy; +1 point for a weak expression of any other emotion or a neutral face; +2 points for a strong expression of any other emotion. This resulted into one categorical variable for each of the items. The online tool did not allow this kind of adjustment of the HDC, therefore, only the SC approach was assessed. The test-retest analysis was assessed by Spearman’s correlation between assessments of EBA emotions in two time points.

**Table 2:** Description of the study sample.

	Total sample		Test-retest subsample		Cortisol subsample	
	N	%	N	%	N	%
Gender						
Male	141	27.0	38	26.0	15	32.6
Female	381	73.0	108	74.0	31	67.4
Age						
15–29 years old	322	61.7	84	57.5	46	100
30–44 years old	116	22.2	34	23.3	-	-
45–59 years old	76	14.6	25	17.1	-	-
60–90 years old	8	1.5	3	2.1	-	-
Marital status						
Single/ Divorced/Widow-widower	344	65.9	95	65.1	46	100
Married	178	34.1	51	34.9	-	-
Highest education achieved						
Elementary school	67	12.8	7	4.8	-	-
Secondary vocational school	18	3.4	4	2.7	-	-
Secondary school with graduation	240	46.0	74	50.7	41	89.1
College / University	197	37.7	61	41.8	5	10.9
Religiosity ^a^						
Believer, member of the church	353	67.6	90	61.6	31	67.4
Believer outside the church	83	15.9	23	15.8	9	19.6
Non-believer	70	13.4	29	19.9	4	8.7
Convinced atheist	16	3.1	4	2.7	2	4.3
Total	522	100	146	100	46	100

Note:

^a^ Independently from church attendance

We analyzed three types of validity: the convergent, discriminant, and criterion validity. For the convergent validity, we used Spearman’s correlations to assess the associations of the EBA-AST with the BSI-53 and of the EBA-SPT with the DSES. Consequently, we assessed the discriminant validity between EBA-AST and EBA-SPT. For the assessment of criterion validity, we used as a first criterion of validity associations with cortisol levels (baseline; 30 minutes after the first measurement, i.e., follow-up; and reactivity, i.e., follow-up level minus baseline level). For the EBA tools, we separately assessed the positive emotion response category (joy) and the other emotions response category. We hypothesized that correlations of positive emotions with cortisol would be negative, and reverse. Before the cortisol analysis, we checked the role of potential confounders (the role of contraceptives and phase of the menstrual cycle) using a linear regression, showing none to confound. Therefore, we decided to proceed without further adjustment to them, just using Spearman’s correlation to assess the association of cortisol levels, primarily with the BSI-53 and with the EBA-AST and consequently with the DSES and with the EBA-SPT.

As a second criterion of validity, we assessed associations of the EBA tools (both SC and HDC), the DSES and the BSI-53 with the Dopen Questionnaire Lie Score using Spearman’s correlation. We hypothesized that weak correlations with Dopen scores would indicate less proneness to social desirability. All analyses were performed using the statistical software package IBM SPSS version 21.

## Results

### Description of the population

The background characteristics of the total sample and the test-retest and cortisol subsamples are presented in Table 2. Of the main sample, 31.8% of the respondents were unable to recognize emotions in the control questions. We have also separately addressed sociodemographic differences regarding gender, age, marital status, education and religious affiliation for each EBA tool. The results of this more detailed comparison are reported in S4 File—Sociodemographic differences. In general, across different EBA tools, significant differences were observed mainly for SC or for HDC of joy, while the HDC for “other emotions” were with one exception non-significant.

### Structure of the EBA tools

The results of Spearman’s correlations of the scores for basic emotions of the EBA tools (see Table 3) showed that all the scores for all emotions except joy were positively correlated for both the explicit and implicit measurement of emotions. For the explicit EBA measure (i.e., SC), joy showed significant negative correlations. The other correlations were rather weak, with the exception of fear-surprise. For the implicit EBA measure (i.e., HDC), joy showed weak negative or no significant correlations with other emotions. However, correlations were in most cases stronger among all the other emotions. Therefore, for some parts of the validity analyses, joy was assessed as a unique response category while all the other emotions were merged together.

**Table 3:** Intercorrelations between EBA-SPT basic emotions (selection and HD counts)—Results of Spearman’s rank order correlation analysis.

		anger	fear	disgust	sadness	surprise	neutral
Selection counts	fear	.092					
	disgust	.151**	.257***				
	sadness	.120*	.143**	.138**			
	surprise	.127**	.343***	.115*	.104*		
	neutral	.104*	-.033	.019	.102*	-.019	
	joy	-.549***	-.379***	-.379***	-.500***	-.407***	-.330***
Hover and display counts	fear	.165**					
	disgust	.258***	.146**				
	sadness	.268***	.142**	.333***			
	surprise	.197***	.272***	.188***	.175***		
	neutral	.273***	.119*	.187***	.266**	.161**	
	joy	-.123*	-.044	-.005	-.149**	-.027	-.065

Notes:

*p < 0.05.

**p < 0.01.

***p < 0.001

The results of the other descriptive analyses showed that the scores per emotion are positively skewed (see Table 4). Table 5 shows correlations between EBA-AST and EBA-SPT.

**Table 4:** Distribution of the scores per emotion: Means, medians, standard deviations (std. dev.) and further measures of variation.

	Mean	95% CI		Median	Std. dev.	Skewness	Kurtosis	Minimum	Maximum
		Lower bound	Upper bound						
**EBA Actual Situation Tool**									
Selection counts									
anger	1.18	1.07	1.28	1	1.24	1.58	4.74	0	9
fear	0.39	0.32	0.46	0	0.82	2.30	5.19	0	5
disgust	0.63	0.54	0.72	0	1.09	2.08	4.99	0	7
sadness	1.42	1.29	1.56	1	1.55	1.06	0.80	0	8
surprise	0.40	0.34	0.47	0	0.76	2.00	3.65	0	4
neutral	0.52	0.45	0.59	0	0.81	2.19	7.00	0	6
joy	6.24	5.96	6.53	6	3.31	0.43	-0.20	0	16
Hover and display counts									
anger	3.67	3.36	3.99	3	3.62	1.90	5.71	0	25
fear	1.70	1.51	1.89	1	2.20	1.68	3.21	0	14
disgust	2.48	2.21	2.74	2	3.07	1.95	4.59	0	17
sadness	4.04	3.69	4.38	3	3.98	1.75	4.74	0	25
surprise	2.08	1.86	2.30	2	2.57	1.86	4.43	0	16
neutral	1.62	1.42	1.82	1	2.31	3.41	18.51	0	21
joy	11.98	11.36	12.59	11	7.20	0.92	0.77	0	38
**EBA Spirituality Tool**									
Selection counts									
anger	1.85	1.67	2.03	1	1.90	1.37	2.12	0	11
fear	1.39	1.21	1.57	0	1.93	1.98	5.97	0	14
disgust	0.75	0.62	0.89	0	1.40	2.96	11.97	0	11
sadness	2.31	2.07	2.56	2	2.57	2.03	6.79	0	18
surprise	1.62	1.45	1.79	1	1.83	1.27	1.45	0	9
neutral	0.78	0.68	0.89	0	1.14	2.21	7.90	0	9
joy	13.64	13.00	14.28	14	6.78	0.19	-0.68	0	31
Hover and display counts									
anger	5.15	4.64	5.67	4	5.49	1.45	2.46	0	32
fear	4.72	4.19	5.25	4	5.59	2.20	9.55	0	48
disgust	3.22	2.79	3.65	2	4.53	2.32	8.46	0	36
sadness	5.63	5.00	6.25	4	6.64	2.03	6.13	0	46
surprise	5.22	4.69	5.75	4	5.60	1.37	1.92	0	30
neutral	2.15	1.83	2.46	0	3.32	3.08	15.69	0	30
joy	21.98	20.87	23.09	21	11.78	0.91	1.97	0	82

^a^ Sum of the number of selections of the emotion as final answer in the online survey; ^b^ Sum of the number of mouse hover events over the emotion; ^c^ Sum of the number of enlarged displays after user clicks on the emotion

**Table 5:** Bivariate associations of scores on the EBA actual situation tool and the EBA Spirituality tool (SC^a^ and HDC^b^) (complete sample).

			**EBA Actual Situation Tool**				**EBA Spirituality Tool**										
							**Complete tool**				**NRS subscale**				**GI subscale**		
			**SC** ^a^		**HDC** ^b^		**SC**		**HDC**		**SC**		**HDC**		**SC**		**HDC**
			**Joy**	**Other** ^c^	**Joy**	**Other**	**Joy**	**Other**	**Joy**	**Other**	**Joy**	**Other**	**Joy**	**Other**	**Joy**	**Other**	**Joy**
**EBA Actual Situation Tool**	**SC**	**Other**	-.87***														
	**HDC**	**Joy**	.74***	-.68***													
		**Other**	-.49***	.51***	-.03												
**EBA Spirituality Tool**	**SC**	**Joy**	.56***	-.52***	.45***	-.28***											
		**Other**	-.52***	.60**	-.45***	.30***	-.87***										
	**HDC**	**Joy**	.44***	-.45***	.56***	.06	.82***	-.75***									
		**Other**	-.29***	.30***	.08	.67***	-.49***	.52***	-.11*								
**NRS subscale**	**SC**	**Joy**	.56***	-.54***	.47***	-.31***	.87***	-.75***	.71***	-.44***							
		**Other**	-.52***	.61***	-.45***	.31***	-.75***	.86***	-.64***	.47***	-.85***						
	**HDC**	**Joy**	.44***	-.46***	.56***	.02	.69***	-.62***	.87***	-.07	.81***	-.72***					
		**Other**	-.30***	.33***	.06	.66***	-.43***	.46***	-.08	.92***	-.51***	.55***	-.16***				
**GI subscale**	**SC**	**Joy**	.42***	-.39***	.33***	-.19***	.92***	-.81***	.76***	-.44***	.60***	-.53***	.48***	-.29**			
		**Other**	-.37***	.43***	-.31***	.22***	-.80***	.90***	-.68***	.47***	-.52***	.58***	-.42***	.30**	-.88**		
	**HDC**	**Joy**	.33***	-.33***	.41***	.07	.78***	-.71***	.89***	-.13**	.50***	-.46***	.56***	-.02	.86**	-.78**	
		**Other**	-.23***	.22***	.10*	.56***	-.47***	.50***	-.13**	.90***	-.30***	.31***	-.001	.67**	-.52**	.57**	-.23**

Notes:

*p < 0.05,

**p < 0.01,

***p < 0.001 (Spearman correlations)

^a^ Selection counts = sum of the number of selections of the emotion as a final answer;

^b^ Hover and display counts = sum of the number of mouse hover events over the emotion + sum of the number of enlarged displays after user clicks on the emotion;

^c^ Other emotions merged

### Psychometric properties

#### Reliability

The results of reliability assessment using the SC are presented in Table 6. We found good internal consistency of the EBA-SPT, and an acceptable internal consistency of the EBA-AST.

**Table 6:** Reliability of the Emotion Based Approach tools.

	Selection counts ^a^		Hover-display counts ^b^	
	Spirituality tool	Actual Situation tool	Spirituality tool	Actual Situation tool
**Internal reliability** ^c^				
**Cronbach’s alpha**	0.82	0.62		
**MIIC** ^d^	0.22	0.17		
**Test-retest reliability** ^e^				
**Anger**	.48***	.31***	.41***	.38***
**Fear**	.45***	.25**	.46***	.23**
**Disgust**	.49***	.35***	**.11**	.27***
**Sadness**	.59***	.37***	.31**	.30***
**Surprise**	.52***	.23**	.53***	.26***
**Neutral**	.35***	**.08**	.22*	**.09**
**Joy**	.83***	.65***	.74***	.49***

Notes:

*p < 0.05,

**p < 0.01,

***p < 0.001

^a^ Sum of the number of selections of the emotion as a final answer in the online survey;

^b^ Sum of the number of mouse hover events over the emotion + sum of the number of enlarged displays after user clicks on the emotion;

^c^ Complete sample;

^d^ Intraclass correlation coefficient;

^e^ Test-retest subsample

In the assessment of the test-retest reliability, we observed mostly similar values of the scores for each emotion in the tools for both SC and HDC. The EBA-SPT showed somewhat higher test-retest correlations than the EBA-AST. Furthermore, we found good reliability for joy, but even very weak reliability for neutral expression in the EBA-AST and for disgust in EBA-SPT.

### Validity

In the further assessment of validity, we assessed the scores for basic emotions of the EBA tools both separately and, based on the results of the correlations between them, merged into an overall cluster, from which only joy was excluded.

#### Convergent and discriminant validity

Table 7 shows the correlations of emotions of the EBA tool, presented both for SC and for HDC, with the BSI-53 and the DSES. Generally, correlations were stronger when the emotions were assessed by SC than by HDC.

**Table 7:** Correlations of the Emotion Based Approach (EBA) actual situation tool and the EBA Spirituality tool (non-religious + god-image items) with the Brief Symptom Inventory and the Daily Spiritual Experience Scale.

		Joy	All other emotions	Joy	All other emotions
**Actual Situation tool**			
**Selection counts** ^a^		**Hover-display counts** ^b^	
**Brief Symptom Inventory**	**Somatization**	-.31***	.34***	-.29***	.12*
	**Obsessive Compulsive**	-.35***	.39***	-.34***	.16**
	**Interpersonal sensitivity**	-.46***	.48***	-.42***	.22***
	**Depression**	-.50***	.50***	-.46***	.20***
	**Anxiety**	-.41***	.45***	-.38***	.17***
	**Hostility**	-.37***	.44***	-.33***	.21***
	**Phobic Anxiety**	-.35***	.39***	-.37***	.13**
	**Paranoid Ideation**	-.43***	.44***	-.37***	.20***
	**Psychoticism**	-.49***	.52***	-.44***	.24***
	**Global Severity Index**	-.52***	.55***	-.47***	.23***
**Daily Spiritual Experience Scale**		**Spirituality tool**			
		**Selection counts**		**Hover-display counts**	
		.57***	-.52***	.48***	-.24***

Notes:

*p < 0.05,

**p < 0.01,

***p < 0.001

^a^ Sum of the number of selections of the emotion as a final answer;

^b^ Sum of the number of mouse hover events over the emotion + sum of the number of enlarged displays after user clicks on the emotion

The discriminant validity of EBA-AST with EBA-SPT was assessed only for selection counts and its value was 0.82.

#### Criterion validity

We assessed criterion validity of the EBA-SPT and EBA-AST compared to standard questionnaires using the associations with cortisol levels and with the Dopen Questionnaire Lie Score as criteria (Table 8). EBA-SPT had weak correlation with cortisol when assessing joy. Both EBA had somewhat stronger significant correlations of the merged other emotions. These associations were stronger for cortisol reactivity, i.e., level differences, than for state at either baseline or follow-up. In all cases, correlations were strongest for the HDC and cortisol reactivity approach regarding these merged other emotions, with most of these correlations being statistically significant. Moreover, for the HDC approach, correlations with social desirability scores were generally weaker and non-significant. More in detail for the EBA-SPT, associations with criteria were better for the NRS than for the GI subscale. Results for the two standard questionnaires (DSES and BSI) were in reverse to those for the EBA HDC approaches, i.e., weak and non-significant for cortisol, and in case of BSI, stronger and significant for social desirability. In summary, results concerning the two criteria were best for the HDC approach, and for the EBA-AST and the EBA-SPT NRS scale were better than for the two standard questionnaires.

**Table 8:** Bivariate associations with cortisol levels and Dopen Questionnaire Lie Score.

			Cortisol ^a^			Social desirability ^b^
			Baseline	Follow-up	Reactivity ^c^	
**EBA Actual Situation Tool**						
	**SC** ^d^	**Joy**	-.17	.18	.22	0.09*
		**Other emotions merged**	.21	-.12	-.21	-0.12*
	**HDC** ^e^	**Joy**	-.15	.10	.13	0.08
		**Other emotions merged**	.34*	-.31*	-.48**	-0.06
**EBA Spirituality Tool**						
	**SC**	**Joy**	-.07	.31*	.26	0.12*
		**Other emotions merged**	.08	-.38*	-.37*	-0.16**
	**HDC**	**Joy**	.02	.26	.18	0.15**
		**Other emotions merged**	.36*	-.41**	-.55***	-0.04
**NRS subscale**	**SC**	**Joy**	-.20	.27	.34*	0.15**
		**Other emotions merged**	.23	-.27	-.38**	-0.15**
	**HDC**	**Joy**	-.10	.18	.20	0.15**
		**Other emotions merged**	.40**	-.39**	-.60***	-0.07
**GI subscale**	**SC**	**Joy**	.02	.22	.13	0.06
		**Other emotions merged**	-.04	-.28	-.19	-0.09
	**HDC**	**Joy**	.05	.16	.06	0.10*
		**Other emotions merged**	.15	-.20	-.23	-0.03
**DSES**			-0.06	0.07	0.10	0.09
**BSI-53**			0.21	0.08	-0.06	-0.17***

Notes:

*p < 0.05,

**p < 0.01,

***p < 0.001

^a^ Cortisol subsample;

^b^ The whole sample;

^c^ Follow-up level—Baseline level;

^d^ Selection counts = Sum of the number of selections of the emotion as a final answer;

^e^ Hover and display counts = Sum of the number of mouse hover events over the emotion + sum of the number of enlarged displays after user clicks on the emotion

## Discussion

The aim of this article was to explore whether our new method, the Emotion Based Approach (EBA), based on the use of a display of photos of basic facial expressions, represents a reliable alternative to classical questionnaires with regards to assessment of attitudes. We found that the EBA tools have acceptable (EBA-AST) to good (EBA-SPT) internal consistency and that specific emotions of the tools differ in their test-retest reliability. An implicit EBA approach (HDC) yielded stronger correlations between the emotions as measured and weaker convergent validity, but higher criterion validity, i.e., as hypothesized stronger correlations with cortisol reactivity and weaker correlations with social desirability scores, than the explicit approach (SC) and standard questionnaires. The EBA thus seems to represent a better approach for measuring attitudes.

We found that the EBA tools had acceptable (EBA-AST) to good (EBA-SPT) internal consistency, but that separate emotions, i.e., response categories, varied in test-retest reliability, from good for joy to very weak for the neutral expression. Generally, research describes low values of reliability analyses for implicit attitude measures [10]. We can also suppose that the lower values of the test-retest reliability for some emotions might reflect the real-life experience of participants, where negative emotions are rarely found as distinct feelings, but rather as their mixture [24]. Therefore, when we consider the nature of the tool, the reliability of EBA-SPT is sufficient.

We further found that the implicit EBA approach (HDC) showed weaker convergent validity (correlations with BSI-53 and DSES) but higher criterion validity than the explicit approach (SC), except for joy. The standard questionnaires were practically not associated with cortisol levels but BSI-53 showed weak correlation with the Lie Score. A first explanation could be that SC resembles standard instruments more than HDC and that these standard instruments are more susceptible to social desirability, more specifically to self-deception. Shedler et al. [7] in their work on mental health (MH) measurement state that standard MH scales appear unable to distinguish between genuine MH and the facade or illusion of MH created by psychological defenses. Social desirability is involved in the interpretation of self-reported items [25]. If participants are not trained in recognizing emotions, both SC and HDC could leave space for respondents to interpret their choice in a socially desirable way, as was also shown in a part of our study. However, our results also suggest that social desirability can still be present if participants are aware that their choice is being assessed (case of selection counts).

Nevertheless, an effort to answer in a socially desirable way may result in a higher number of preliminary choices (i.e., HDC) or simply a longer time before choosing the final option. This explanation might be partly supported by many well-documented findings from studies on the Word-Association Test [26]. In this test researchers suggest that the longer the reaction time, i.e., the time between the presentation of a stimulus and the occurrence of a response, the higher is the rationalization of the answer. This possibility may also correspond to the presumptions of the newer Implicit Association Test [9]. A last explanation for HDC showing stronger associations with cortisol levels than SC is that a non-specific impairment of cognitive functioning due to acute or chronic stress could manifest itself as a difficulty to make a complex choice.

In our study, almost one-third of the participants failed to identify three basic emotions in the control set of questions. However, excluding these participants from the analyses did not improve any finding. These findings suggest that the ability to verbally label the emotion is not necessarily associated with the non-conscious ability to read and express it. This hypothesis is supported by studies documenting discrimination and imitation of facial expressions by neonates [27] and toddlers´ understanding of the emotions of their peers [28], as these children are able to react to the emotions of others even though they are not able to describe them verbally. We can therefore conclude that the inability to label the emotion is no objection to participation in this kind of test, but even more that this may represent an advantage in research on attitudes by limiting the response bias. However, further research is needed to show if this applies in general to people with seriously high emotional unawareness.

Our last findings regarded the differences between outcomes of the concrete EBA tools (i.e., the various sets of items) because of differences in the wording of the questions. We found that the SPT-NRS was more strongly and significantly correlated with cortisol levels than the SPT-GI and that responses on all the EBA tools differed in their associations with sociodemographic variables. These differences can be interpreted as that the theme and the wording of the items are important and that respondents did not simply project their actual mood onto the test, but that this approach assesses also more stable feelings related to different areas of assessment. The question remains as to what degree the actual mood of respondents interferes with their choices.

### Strengths and limitations

This study has several important strengths. The most important is that it offers a new and easily administrable approach to measuring implicit attitudes. Second, it offers two concrete tools, the EBA Spirituality tool for spirituality assessment and the EBA Actual Situation tool for measuring the actual distress. Moreover, it gives instructions for creating other EBA tools, designed according to research purposes. Our study also presents two possible approaches, more explicit and implicit, of using EBA and compares the results of their use. Third, we present both convergent and criterion validity assessments of the new tools.

A limitation of our study relates to the way of scoring emotions, because a decision about scoring the intensity of the emotions is to a certain degree arbitrary, unless using prototypical faces with two computer generated levels of emotion expressions. A second limitation is the relatively low number of respondents in the cortisol assessment study. A third limitation is that the whole survey was administered in home conditions, which means we could not control possible disturbing elements. Moreover, based on our study sample, we were not able to determine the validity of the EBA approach for respondents with different levels of emotional intelligence. Thus, more research is needed on this topic. As for concrete tools, the EBA AST might miss some aspects that could also contribute to one’s well-being, and if disrupted, could cause a higher level of stress, e.g., the economic aspects and one’s standard of living or one’s living environment. Therefore, future studies could include additional items in an EBA tool that register potential actual distress in a better way.

### Implications

This study offers a new method, the EBA, which is suitable for use both in qualitative and quantitative research. We suggest that an assessment of the selection process (HDC) may represent a better way of measurement than the mere selection of a face, and so that an online tool is the most suitable way of administration. However, our results indicate that even the paper-pencil administration might lead to satisfactory results.

Future research should assess whether including a male face for male participants (or including a choice) makes a distinction concerning their choices and should also compare the results gained by paper-pencil administration with those obtained as SC through the online tool. Furthermore, based on our results and the nature of specific emotions, the exclusion of a neutral and/or surprised face could be considered. Moreover, other possible ways of scoring the EBA tool should be explored. With regards to the items of the tools, for further use, the EBA AST should be enriched by items covering other aspects that could also contribute to one’s well-being and could influence one’s level of stress. In order to support the proposed link between one´s choices in the EBA tools and a physiological state of the body, future research should assess associations of the EBA scores with other physiological measures (e.g., EEG and biofeedback) in controlled experimental conditions.

## Conclusion

We found that the more implicit approach, i.e., using the display of basic emotions instead of a classical verbal choice, represents a more reliable approach for measuring attitudes than standard questionnaires. Moreover, assessing also the selection process (HDC) seems to offer even better insight into the participants’ deeper feelings. Our EBA method therefore represents a useful approach that helps to lower the effects of social desirability.

## Supporting information

S1 File Instructions for the EBA tools and their items. (DOCX)

S2 File Control questions on reading emotions. (DOCX)

S3 File Results of the pilot study. (DOCX)

S4 File Sociodemographic differences. (DOCX)

S5 File A new approach to measuring implicit attitudes—Main sample. Database for the analyses in the main text.
(SAV)

S6 File A new approach to measuring implicit attitudes—Pilot sample. Database for the analyses described in S3 File.
(SAV)
