# Supplementary material for: Using photos of basic facial expressions as a new approach to measuring implicit attitudes
Source: PLoS One. 2021 May 13;16(5):e0250922. doi: 10.1371/journal.pone.0250922 (PMC8118344; doi:10.1371/journal.pone.0250922)
Supplement: S1 File — (DOCX) [file pone.0250922.s001.docx]

**S1 File. Instructions for the EBA tools and their items.**

**Introductory instruction:**

The introductory instruction was worded as follows:

*“Please make sure that you can fill out this part of the questionnaire (approx. 10-20 min)^[[1]](#footnote-1)^ without time delays and in an undisturbed environment. Try not to think about your choice and let yourself be guided by your feelings. If it helps, you can close your eyes for a while and tune in to the given term. Faces will automatically enlarge when you move the cursor, and when you click on the picture, you will be able to view the image at its maximum size and either confirm the selection or close it and look at another.”*

For every item the text was worded as follows:

*“Choose the face that best describes what you feel when you say: *wording of the item*. Try not to think [identical text as above] and look at another.”*

**Questionnaire items:**

**EBA Spirituality Tool**

**Non-religious items (SPT-NR)**

- meaning of life
- me and the world
- my past
- my future
- my spiritual life
- the aim of my life
- forgiveness
- engagement for others

**God-Image items (SPT-GI)**

- God
- prayer
- God’s will
- God’s closeness
- I can hear God talking about me
- meeting with God at the end of my life
- alone with God
- God in my life

**Actual Situation Tool (AST)**

- how am I
- today
- people around me
- my life
- my work
- my relationships
- my needs
- my health

1. This time span regards the time needed to fill in the three EBA tools. Filling in the whole online survey took over 30 minutes as a median. [↑](#footnote-ref-1)
