# Supplementary material for: Using photos of basic facial expressions as a new approach to measuring implicit attitudes
Source: PLoS One. 2021 May 13;16(5):e0250922. doi: 10.1371/journal.pone.0250922 (PMC8118344; doi:10.1371/journal.pone.0250922)
Supplement: S2 File — (DOCX) [file pone.0250922.s002.docx]

**S2 File.** **Control questions on reading emotions.**

**Wording of the questions**

*The photos of basic emotions used in the research were blurred in the following figures in order to protect the rights of the copyright holder and are used only for illustrative purposes.*


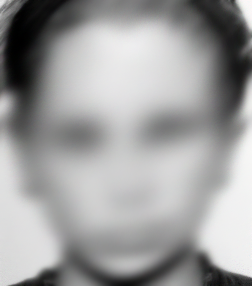


**1. In your opinion, this girl is probably:**

a) Celebrating a birthday with a friend

b) Enjoying watching a romantic movie

c) She found out that she was robbed in the store


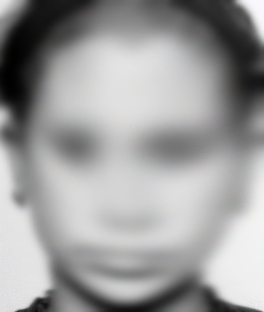


**2. In your opinion, this girl is probably:**

a) Watching horror on TV

b) She found out that her dog had died

c) She just won the lottery

**
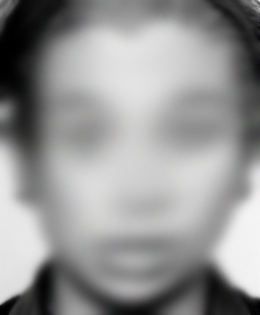
**

**3. In your opinion, this girl is probably:**

a) Cleaning the bathroom

b) She bought a nice handbag

c) She sees an unknown man following her on the street at night

**Correct answers: 1c, 2b, 3c**

**Comparison of the groups of respondents who were able to recognize emotions and those who were not**

In an additional assessment, we have compared the results of the assessment of cortisol levels for the whole group (n=46; see Supplementary Table 2/1) and for a subsample of 36 respondents (33 for Spirituality Tool – God image subscale; see Supplementary Table 2/2) who were able to recognize emotions. Below, these assessments are shown for the whole group and separately for only the respondents who could discriminate emotions:

**Supplementary Table 2/1. Assessment performed on the whole group (see Table 8 of the main document).**

|  |  |  | **Cortisol ^a^** | | |
| --- | --- | --- | --- | --- | --- |
|  |  |  | **Baseline** | **Follow-up** | **Reactivity ^c^** |
| **EBA Actual Situation Tool** | |  |  |  |  |
|  | **SC ^d^** | **Joy** | -.17 | .18 | .22 |
|  |  | **Other emotions merged** | .21 | -.12 | -.21 |
|  | **HDC ^e^** | **Joy** | -.15 | .10 | .13 |
|  |  | **Other emotions merged** | **.34^*^** | **-.31^*^** | **-.48^**^** |
| **EBA Spirituality Tool** | |  |  |  |  |
|  | **SC** | **Joy** | -.07 | **.31^*^** | .26 |
|  |  | **Other emotions merged** | .08 | **-.38^*^** | **-.37^*^** |
|  | **HDC** | **Joy** | .02 | .26 | .18 |
|  |  | **Other emotions merged** | **.36^*^** | **-.41^**^** | **-.55^***^** |
| **NRS subscale** | **SC** | **Joy** | -.20 | .27 | **.34^*^** |
|  |  | **Other emotions merged** | .23 | -.27 | **-.38^**^** |
|  | **HDC** | **Joy** | -.10 | .18 | .20 |
|  |  | **Other emotions merged** | **.40^**^** | **-.39^**^** | **-.60^***^** |
| **GI subscale** | **SC** | **Joy** | .02 | .22 | .13 |
|  |  | **Other emotions merged** | -.04 | -.28 | -.19 |
|  | **HDC** | **Joy** | .05 | .16 | .06 |
|  |  | **Other emotions merged** | .15 | -.20 | -.23 |

Notes: *p < 0.05, **p < 0.01, ***p < 0.001

^a^ Cortisol subsample; ^b^ Follow-up level – Baseline level; ^c^ SC - Selection counts = Sum of the number of selections of the emotion as a final answer; ^d^ HDC - Hover and display counts = Sum of the number of mouse hover events over the emotion + sum of the number of enlarged displays after user clicks on the emotion; EBA - Emotion Based Approach; NRS - Non-religious spirituality; GI - God-Image

**Supplementary Table 2/2. Assessment performed on a subsample of respondents who were able to recognize emotions.**

|  |  |  | **Cortisol ^a^** | | |
| --- | --- | --- | --- | --- | --- |
|  |  |  | **Baseline** | **Follow-up** | **Reactivity ^b^** |
| **EBA Actual Situation Tool** | |  |  |  |  |
|  | **SC ^c^** | **Joy** | -.21 | .11 | .21 |
|  |  | **Other emotions merged** | .31 | -.10 | -.24 |
|  | **HDC ^d^** | **Joy** | -.28 | -.02 | .12 |
|  |  | **Other emotions merged** | **.41^*^** | -.21 | **-.42^*^** |
| **EBA Spirituality Tool** | |  |  | -.23 | .14 |
|  | **SC** | **Joy** | .18 | -.26 | -.32 |
|  |  | **Other emotions merged** | -.15 | .09 | .15 |
|  | **HDC** | **Joy** | **.48^**^** | **-.39^*^** | **-.59^**^** |
|  |  | **Other emotions merged** | **-.38^*^** | .09 | .28 |
| **NRS subscale** | **SC** | **Joy** | **.37^*^** | -.14 | -.30 |
|  |  | **Other emotions merged** | -.27 | -.06 | .10 |
|  | **HDC** | **Joy** | **.46^**^** | **-.39^*^** | **-.58^**^** |
|  |  | **Other emotions merged** | -.10 | .14 | .18 |
| **GI subscale** | **SC** | **Joy** | .09 | -.18 | -.23 |
|  |  | **Other emotions merged** | -.04 | .09 | .10 |
|  | **HDC** | **Joy** | .28 | -.13 | -.29 |
|  |  | **Other emotions merged** | -.21 | .11 | .21 |

Notes: *p < 0.05, **p < 0.01, ***p < 0.001

^a^ Cortisol subsample; ^b^ Follow-up level – Baseline level; ^c^ SC - Selection counts = Sum of the number of selections of the emotion as a final answer; ^d^ HDC - Hover and display counts = Sum of the number of mouse hover events over the emotion + sum of the number of enlarged displays after user clicks on the emotion; EBA - Emotion Based Approach; NRS - Non-religious spirituality; GI - God-Image

As can be seen, discarding the respondents who were not able to discern emotions did not lead to better figures, rather some significant findings were lost due to the lower power of the analysis. Therefore, we do not recommend excluding these participants and in the main document, we present only the analyses performed on the whole sample.
