# Supplementary material for: Using photos of basic facial expressions as a new approach to measuring implicit attitudes
Source: PLoS One. 2021 May 13;16(5):e0250922. doi: 10.1371/journal.pone.0250922 (PMC8118344; doi:10.1371/journal.pone.0250922)
Supplement: S3 File — (DOCX) [file pone.0250922.s003.docx]

**S3 File. Results of the pilot study.**

## Description of the population

Supplementary Table 1 provides basic sociodemographic characteristics of the pilot sample.

**Supplementary Table 1. Description of the pilot study sample**

|  | Total sample | | Cortisol  subsample | |
| --- | --- | --- | --- | --- |
|  |  |  |  |  |
|  | N | % | N | % |
| **Gender** |  |  |  |  |
| Male | 39 | 22.3 | 26 | 25.2 |
| Female | 136 | 77.7 | 77 | 74.8 |
| **Age** |  |  |  |  |
| 15-29 years old | 131 | 74.9 | 103 | 100 |
| 30-44 years old | 27 | 15.4 | - | - |
| 45-59 years old | 17 | 9.7 | - | - |
| 60-90 years old | - | - | - | - |
| **Marital status** |  |  |  |  |
| Single/Divorced/Widow-widower | 138 | 78.9 | 102 | 99.0 |
| Married | 37 | 21.1 | 1 | 1.0 |
| **Highest education achieved** |  |  |  |  |
| Elementary school | 2 | 1.1 | - | - |
| Secondary vocational school | 1 | 0.6 | - | - |
| Secondary school with graduation | 106 | 60.6 | 82 | 79.6 |
| College / University | 66 | 37.7 | 21 | 20.4 |
| **Religiosity ^a^** |  |  |  |  |
| Believer, member of the church | 129 | 73.7 | 75 | 72.8 |
| Believer outside the church | 26 | 14.9 | 17 | 16.5 |
| Non-believer | 19 | 10.9 | 10 | 9.7 |
| Convinced atheist | 1 | 0.6 | 1 | 1.0 |
| **Total** | 175 | 100 | 103 | 100 |

Note: ^a^ Independently from church attendance

## Structure of the EBA-SPT

The results of Spearman’s correlations of the scores for basic emotions of the primary EBA tools (see Supplementary Table 2) showed that for SC, all the scores for all emotions except neutral and joy were mostly positively correlated. However, the correlations were rather weak. Regarding HD counts, all the emotions were strongly positively correlated.

**Supplementary Table 2: Intercorrelations between EBA-SPT basic emotions (selection and HD counts) – results of Spearman’s rank order correlation analysis.**

|  |  | anger | fear | disgust | sadness | surprise | neutral |  |
| --- | --- | --- | --- | --- | --- | --- | --- | --- |
| Selection counts | fear | .046 |  |  |  |  |  |  |
|  | disgust | .**240**** | -.028 |  |  |  |  |  |
|  | sadness | .**267**** | .**176*** | .070 |  |  |  |  |
|  | surprise | .**190*** | .109 | .**178*** | .**217**** |  |  |  |
|  | neutral | -.**237**** | -.016 | -.**243**** | -.077 | **-.198*** |  |  |
|  | joy | -.**304***** | -.**282***** | -.**170*** | **-.374***** | **-.364***** | **-.219***** |  |
| Hover and display counts | fear | **.690***** |  |  |  |  |  |  |
|  | disgust | **.755***** | **.576***** |  |  |  |  |  |
|  | sadness | **.737***** | **.667***** | **.693***** |  |  |  |  |
|  | surprise | **.662***** | **.638***** | **.600***** | **.725***** |  |  |  |
|  | neutral | **.636***** | **.741***** | **.576***** | **.621***** | **.570***** |  |  |
|  | joy | **.558***** | **.634***** | **.579***** | **.588***** | **.521***** | **.580***** |  |

Notes: *p < 0.05. **p < 0.01. ***p < 0.001

## Validity

**Criterion validity.** We assessed the criterion validity of the pilot EBA-SPT using the associations with cortisol levels as criterion (Supplementary Table 3). The only significant associations were observed for selection counts, a majority of them regarded joy.

**Supplementary Table 3. Bivariate associations of the pilot version of the EBA Spirituality Tool with cortisol levels**

|  |  |  | **Cortisol ^a^** | | |
| --- | --- | --- | --- | --- | --- |
|  |  |  | **Baseline** | **Follow-up** | **Reactivity ^b^** |
| **EBA-SPT** | |  |  |  |  |
|  | **SC ^c^** | **Joy** | -.156 | .174 | **.235*** |
|  |  | **Other emotions merged** | -.018 | -.159 | -.110 |
|  | **HDC ^d^** | **Joy** | -.063 | .045 | .068 |
|  |  | **Other emotions merged** | .100 | -.041 | -.143 |
| **NRS**  **subscale** | **SC** | **Joy** | -.154 | .157 | **.230*** |
|  |  | **Other emotions merged** | .154 | -.157 | **-.230*** |
|  | **HDC** | **Joy** | -.066 | -.010 | .023 |
|  |  | **Other emotions merged** | .123 | -.077 | **-.209*** |
| **GI subscale** | **SC** | **Joy** | -.106 | .154 | .184 |
|  |  | **Other emotions merged** | -.114 | -.156 | -.038 |
|  | **HDC** | **Joy** | -.040 | .095 | .121 |
|  |  | **Other emotions merged** | -.003 | -.043 | -.030 |

Notes: *p < 0.05, **p < 0.01, ***p < 0.001

^a^ Cortisol subsample; ^b^ Follow-up level – Baseline level; ^c^ Selection counts = Sum of the number of selections of the emotion as a final answer; ^d^ Hover and display counts = Sum of the number of mouse hover events over the emotion + sum of the number of enlarged displays after user clicks on the emotion
