# Supplementary material for: Using photos of basic facial expressions as a new approach to measuring implicit attitudes
Source: PLoS One. 2021 May 13;16(5):e0250922. doi: 10.1371/journal.pone.0250922 (PMC8118344; doi:10.1371/journal.pone.0250922)
Supplement: S4 File — (DOCX) [file pone.0250922.s004.docx]

**S4 File. Sociodemographic differences.**

**Results**

We separately addressed sociodemographic differences regarding gender, age, marital status, education and religious affiliation for each EBA tool (see Supplementary Tables 4/1-4/3). Regarding gender differences, in the selection counts we found small and sometimes (in three cases) significant differences between genders: women tend to show higher values for joy and lower for other emotions than men. However, these differences were with one exception significant only in EBA SPT-NR. For the HD counts the tendency was similar; however, the results were significant only for joy in SPT-NR.

Moreover, we found significant differences by education and age, but without a clear trend that would be observable through all the tools. However, we found an increasing prevalence of joy and decreasing prevalence of other emotions at a higher educational level, except for HD counts of “other emotions”.

In most cases, respondents living in a marriage or in a partner relationship showed significantly more joy and significantly less of other emotions compared to singles. However, there were no significant differences in HD counts of “other emotions”. Regarding religious affiliation, we found in the selection counts of SPT-NR and SPT-GI more joy and less other emotions for members of the church than for other groups.

**Supplementary** **Table 4/1. Description of the study sample: Actual Situation tool**

|  | **Selection count** | | | | | | **Hover and display count** | | | | | | |  |  |
| --- | --- | --- | --- | --- | --- | --- | --- | --- | --- | --- | --- | --- | --- | --- | --- |
|  | **Joy** | | | **Other emotions merged** | | | | **Joy** | | | **Other emotions merged** | | | |  |
|  | **Mean** | **Std. Dev.** | p | **Mean** | **Std. Dev.** | p | | **Mean** | **Std. Dev.** | p | **Mean** | **Std. Dev.** | p | | |
| Gender |  |  |  |  |  |  | |  |  |  |  |  |  | | |
| Female | 6.4 | 3.42 | *n.s.* | 4.4 | 2.78 | p<0.05 | | 12.3 | 7.49 | *n.s.* | 15.3 | 12.04 | *n.s.* | | |
| Male | 5.8 | 2.96 |  | 5.0 | 2.59 |  |  | 11.0 | 6.29 |  | 16.4 | 10.69 |  |  |  |
| Age |  |  |  |  |  |  | |  |  |  |  |  |  | | |
| 15-29 years old | 6.0 | 3.25 | p<0.05 (1-3*) | 5.0 | 2.67 | p<0.001 (1-3***; 2-3*) | | 11.1 | 6.78 | p<0.001 (1-3***) | 15.3 | 10.08 | *n.s.* | | |
| 30-44 years old | 6.1 | 3.22 |  | 4.3 | 2.72 |  |  | 12.4 | 7.01 |  | 16.4 | 13.74 |  |  |  |
| 45-59 years old | 7.3 | 3.39 |  | 3.1 | 2.53 |  |  | 15.1 | 8.18 |  | 15.6 | 14.56 |  |  |  |
| 60-90 years old | 6.5 | 4.41 |  | 3.9 | 3.04 |  |  | 12.1 | 8.97 |  | 14.5 | 11.25 |  |  |  |
| Marital status |  |  |  |  |  |  | |  |  |  |  |  |  | | |
| Single/ Divorced/Widow-widower | 5.9 | 3.17 | p<0.01 | 4.9 | 2.67 | p<0.001 | | 10.9 | 6.53 | p<0.001 | 15.3 | 9.65 | *n.s.* | | |
| Married | 6.9 | 3.47 |  | 3.8 | 2.72 |  |  | 14.1 | 7.93 |  | 16.2 | 14.88 |  |  |  |
| Highest education achieved |  |  |  |  |  |  | |  |  |  |  |  |  | | |
| Elementary school | 5.6 | 2.68 | *n.s.* | 5.7 | 2.51 | p<0.001 (1-3**; 1-4***) | | 10.6 | 6.56 | *n.s.* | 13.7 | 8.20 | *n.s.* | | |
| Secondary vocational school | 5.8 | 3.42 |  | 4.9 | 2.40 |  |  | 10.3 | 6.19 |  | 14.1 | 7.92 |  |  |  |
| Secondary school with graduation | 6.4 | 3.32 |  | 4.5 | 2.72 |  |  | 11.5 | 6.47 |  | 15.1 | 10.04 |  |  |  |
| College | 6.3 | 3.47 |  | 4.1 | 2.75 |  |  | 13.1 | 8.15 |  | 17.0 | 14.45 |  |  |  |
| Religiosity^a^ |  |  |  |  |  |  | |  |  |  |  |  |  | | |
| Believer, member of the church | 6.4 | 3.45 | *n.s.* | 4.4 | 2.79 | *n.s.* | | 12.6 | 7.62 | *n.s.* | 16.2 | 12.63 | *n.s.* | | |
| Believer outside the church | 5.7 | 3.05 |  | 4.9 | 2.69 |  |  | 10.8 | 6.46 |  | 14.7 | 9.59 |  |  |  |
| Non-believer | 6.2 | 3.03 |  | 4.8 | 2.67 |  |  | 11.1 | 5.80 |  | 14.30 | 9.55 |  |  |  |
| Convinced atheist | 6.4 | 2.42 |  | 5.4 | 2.00 |  |  | 9.1 | 5.28 |  | 11.3 | 6.34 |  |  |  |

Note: ^a^ Independently from church attendance

**Supplementary** **Table 4/2. Description of the study sample: Spirituality tool – Non-religious subscale**

|  | **Selection count** | | | | | | **Hover and display count** | | | | | |
| --- | --- | --- | --- | --- | --- | --- | --- | --- | --- | --- | --- | --- |
|  | **Joy** | | | **Other emotions merged** | | | **Joy** | | | **Other emotions merged** | | |
|  | **Mean** | **Std. Dev.** | p | **Mean** | **Std. Dev.** | p | **Mean** | **Std. Dev.** | p | **Mean** | **Std. Dev.** | p |
| Gender |  |  |  |  |  |  |  |  |  |  |  |  |
| Female | 6.6 | 3.43 | p<0.05 | 4.6 | 2.87 | p<0.05 | 10.9 | 6.39 | p<0.05 | 14.2 | 9.70 | *n.s.* |
| Male | 5.8 | 3.09 |  | 5.1 | 2.70 |  | 9.5 | 5.62 |  | 14.4 | 8.23 |  |
| Age |  |  |  |  |  |  |  |  |  |  |  |  |
| 15-29 years old | 6.0 | 3.25 | p<0.001 (1-3***) | 5.3 | 2.61 | p<0.001 (1-2*; 1-3***; 2-3**) | 9.5 | 5.49 | p<0.001 (1-3***; 2-3*) | 14.8 | 8.69 | p<0.05 (1-3*) |
| 30-44 years old | 6.5 | 3.57 |  | 4.5 | 3.06 |  | 11.3 | 7.17 |  | 14.0 | 9.68 |  |
| 45-59 years old | 7.7 | 3.06 |  | 3.0 | 2.72 |  | 13.6 | 6.30 |  | 12.5 | 11.32 |  |
| 60-90 years old | 6.8 | 4.43 |  | 4.3 | 2.49 |  | 12.0 | 8.11 |  | 13.3 | 5.78 |  |
| Marital status |  |  |  |  |  |  |  |  |  |  |  |  |
| Single/ Divorced/Widow-widower | 5.9 | 3.33 | p<0.001 | 5.2 | 2.75 | p<0.001 | 9.6 | 5.72 | p<0.001 | 14.4 | 8.62 | *n.s.* |
| Married | 7.2 | 3.28 |  | 3.8 | 2.76 |  | 12.4 | 6.71 |  | 13.8 | 10.54 |  |
| Highest education achieved |  |  |  |  |  |  |  |  |  |  |  |  |
| Elementary school | 5.4 | 3.33 | p<0.05 (1-4*) | 6.3 | 2.85 | p<0.001 (1-3**; 1-4***; 3-4*) | 8.3 | 5.65 | p<0.001 (1-4***; 3-4**) | 13.9 | 8.34 | *n.s.* |
| Secondary vocational school | 6.1 | 3.42 |  | 5.3 | 2.66 |  | 9.9 | 6.32 |  | 14.4 | 9.06 |  |
| Secondary school with graduation | 6.2 | 3.30 |  | 4.8 | 2.67 |  | 9.9 | 5.65 |  | 14.3 | 8.84 |  |
| College | 6.9 | 3.37 |  | 4.1 | 2.84 |  | 12.1 | 6.71 |  | 14.3 | 10.23 |  |
| Religiosity^a^ |  |  |  |  |  |  |  |  |  |  |  |  |
| Believer, member of the church | 6.7 | 3.45 | p<0.05 (1-3*) | 4.4 | 2.74 | p<0.001  (1-3**; 1-4*) | 11.2 | 6.47 | p<0.01  (1-3*; 1-4*) | 14.3 | 9.46 | *n.s.* |
| Believer outside the church | 6.0 | 3.08 |  | 5.0 | 2.76 |  | 10.0 | 5.52 |  | 14.2 | 9.24 |  |
| Non-believer | 5.4 | 3.11 |  | 5.8 | 3.05 |  | 8.8 | 5.54 |  | 14.3 | 9.13 |  |
| Convinced atheist | 5.4 | 2.73 |  | 6.4 | 2.73 |  | 6.5 | 3.44 |  | 11.5 | 7.48 |  |

Note: ^a^ Independently from church attendance

**Supplementary** **Table 4/3. Description of the study sample: Spirituality tool – God image subscale**

|  | **Selection count** | | | | | | **Hover and display count** | | | | | |
| --- | --- | --- | --- | --- | --- | --- | --- | --- | --- | --- | --- | --- |
|  | **Joy** | | | **Other emotions merged** | | | **Joy** | | | **Other emotions merged** | | |
|  | **Mean** | **Std. Dev.** | p | **Mean** | **Std. Dev.** | p | **Mean** | **Std. Dev.** | p | **Mean** | **Std. Dev.** | p |
| Gender |  |  |  |  |  |  |  |  |  |  |  |  |
| Female | 7.2 | 4.13 | *n.s.* | 4.0 | 3.15 | *n.s.* | 11.2 | 7.05 | *n.s.* | 12.9 | 8.65 | *n.s.* |
| Male | 6.8 | 4.27 |  | 4.6 | 3.24 |  | 10.7 | 6.46 |  | 13.5 | 8.90 |  |
| Age |  |  |  |  |  |  |  |  |  |  |  |  |
| 15-29 years old | 6.5 | 4.08 | p<0.01 (1-3*) | 4.8 | 3.18 | p<0.001 (1-3***; 2-3*) | 9.8 | 6.08 | p<0.001 (1-3***) | 13.5 | 8.44 | *n.s.* |
| 30-44 years old | 7.5 | 4.28 |  | 3.9 | 3.08 |  | 11.8 | 7.50 |  | 12.6 | 8.34 |  |
| 45-59 years old | 8.2 | 3.94 |  | 2.6 | 2.74 |  | 14.0 | 7.54 |  | 12.0 | 10.12 |  |
| 60-90 years old | 9.4 | 4.65 |  | 2.6 | 2.30 |  | 16.3 | 7.95 |  | 14.3 | 9.59 |  |
| Marital status |  |  |  |  |  |  |  |  |  |  |  |  |
| Single/ Divorced/Widow-widower | 6.4 | 4.08 | p<0.001 | 4.7 | 3.13 | p<0.001 | 9.7 | 6.25 | p<0.001 | 13.6 | 8.73 | *n.s.* |
| Married | 8.2 | 4.08 |  | 3.3 | 3.04 |  | 13.2 | 7.37 |  | 12.1 | 8.59 |  |
| Highest education achieved |  |  |  |  |  |  |  |  |  |  |  |  |
| Elementary school | 6.0 | 4.35 | p<0.05 | 5.2 | 3.14 | p<0.01  (1-4**) | 8.1 | 5.99 | p<0.01  (1-3*; 1-4***) | 12.5 | 9.08 | *n.s.* |
| Secondary vocational school | 6.1 | 3.83 |  | 4.9 | 2.89 |  | 9.3 | 5.72 |  | 12.7 | 6.71 |  |
| Secondary school with graduation | 7.0 | 4.10 |  | 4.3 | 3.19 |  | 10.6 | 6.23 |  | 13.3 | 8.52 |  |
| College | 7.6 | 4.14 |  | 3.7 | 3.12 |  | 12.5 | 7.56 |  | 12.9 | 8.98 |  |
| Religiosity^a^ |  |  |  |  |  |  |  |  |  |  |  |  |
| Believer, member of the church | 7.7 | 4.05 | p<0.001 | 3.7 | 2.93 | p<0.001 | 11.9 | 6.82 | p<0.001 | 12.7 | 8.79 | *n.s.* |
| Believer outside the church | 4.6 | 3.68 |  | 6.1 | 3.45 |  | 7.2 | 5.92 |  | 14.6 | 8.22 |  |
| Non-believer |  |  |  |  |  |  |  |  |  |  |  |  |
| Convinced atheist |  |  |  |  |  |  |  |  |  |  |  |  |

Note: ^a^ Independently from church attendance

**Discussion**

We found that compared to men, women tended to report more positive emotions. These findings seem to contradict a recent meta-analysis of Batz-Barbarich, Tay [1], who did not find any significant differences in well-being by gender. They, however, also noted that even previous meta-analyses differed in their conclusions on this issue. An explanation may be that this differs by EBA tools, as three of the four significant results were found for SPT-NR. Then an explanation could also be a more positive attitude of women to spirituality or a spiritual side of life in general. This gender difference is also found in studies reporting the higher spirituality of Czech women compared to men [2, 3].

We further found a higher prevalence of joy for respondents with a higher educational level or with a partner, and for religious people who went to church, in all the EBA tools. Regarding education, this may indicate a general characteristic, which is in line with studies describing a correlation between school education and happiness in life [4]. The finding regarding respondents with a partner is in line with the findings of Lehmann and Tuinman [5]. Regarding religious respondents who were members of a church and showed more positive emotions, our findings add to the ongoing debate about the move from religious structures to individual spiritual experience [6]. Our findings can be interpreted that members of a church show more positive emotions towards spiritual issues than non-members. Another explanation is that most of our significant findings regarded SC or HDC of joy and only one HDC of “other emotions”. However, these HDC were most strongly correlated with cortisol levels, while not correlated with social desirability. On the contrary, other measures showed a less clear or even opposite pattern. So, an explanation for the above described findings may also regard social desirability, especially its self-deception component. More research is needed to distinguish genuine socioeconomic differences from e.g., playing a role that would in one´s eyes correspond to one´s status and life situation.

**References:**

1. Subjective Well-Being: Estimating Effect Sizes and Associations With Gender Inequality. Psychol Sci. 2018; 29(9): 1491-1503. doi:10.1177/0956797618774796
2. Koenig HG. Concerns about measuring "Spirituality" in research. J Nerv Ment Dis. 2008; 196(5), 349-355. doi:10.1097/NMD.0b013e31816ff796
3. Lehmann V, Tuinman MA, Braeken J, Vingerhoets A, Sanderman R, Hagedoorn M. Satisfaction with Relationship Status: Development of a New Scale and the Role in Predicting Well-Being. J Happiness Stud. 2015; 16(1): 169-184. doi:10.1007/s10902-014-9503-x
4. Malinakova K, Trnka R, Sarnikova G, Smekal V, Furstova J, Tavel P. Psychometric evaluation of the Daily Spiritual Experience Scale (DSES) in the Czech environment. Cesk Psychol. 2018; 62(Supplement 1):100–13.
5. Oreopoulos P, Salvanes KG. Priceless: The Nonpecuniary Benefits of Schooling. J Econ Perspect. 2011; 25(1), 159-184. doi:10.1257/jep.25.1.159
6. Sarnikova G, Malinakova K, Furstova J, Dubovska E, Tavel P. Psychometric evaluation of the Functional Assessment of Chronic Illness Therapy-Spiritual Well-Being (FACIT-Sp) Scale in the Czech environment. Cesk Psychol. 2018; 62, 114-128.
